# Use of Objective Structured Clinical Examination (OSCE) in a hybrid digital / in-person training for hormonal IUD in Nigeria: findings and applications of the approach

**DOI:** 10.12688/gatesopenres.14695.1

**Published:** 2023-09-12

**Authors:** Ezechukwu Nwokoma, Helen Anyasi, Samantha Archie, Chinedu Onyezobi, Funmilola OlaOlorun, Jennifer Anyanti, Anthony Nwala, Kayode Afolabi, Kristen Little, Eden Demise, Kendal Danna, Kate Rademacher, Marya Plotkin

**Affiliations:** 1 Society for Family Health Nigeria, Abuja, Federal Capital Territory, Nigeria; 2 FHI 360 Nigeria, Abuja, Federal Capital Territory, Nigeria; 3 FHI 360, Durham, North Carolina, USA; 4 College of Medicine, University of Ibadan, Evidence for Sustainable Human Development Systems in Africa, Ibadan, Oyo, Nigeria; 5 Reproductive Health Division, Nigeria Federal Ministry of Health, Abuja, Federal Capital Territory, Nigeria; 6 Population Services International, Washington, District of Columbia, USA

**Keywords:** Knowledge assessments, skills assessments, Objective Structured Clinical Examination (OSCE), digital training, hormonal IUD, Nigeria

## Abstract

**Background**: The hormonal intrauterine device, a long-acting reversible contraceptive method, is being introduced to pilot sites in the private and public sector in Nigeria by the Nigerian Federal Ministry of Health since 2019. To inform training of health care providers, a study was conducted on a hybrid digital and in-person training which utilized Objective Structured Clinical Examination (OSCE) to assess competency of provider trainees. This study represents one of few documented experiences using OSCE to assess the effectiveness of a digital training.

**Methods**: From September – October 2021, in Enugu, Kano and Oyo states of Nigeria, 62 health care providers from public and private sector health facilities were trained in hormonal IUD service provision using a hybrid digital / in-person training approach. Providers, who were skilled in provision of copper IUD, underwent a didactic component using digital modules, followed by an in-person practicum, and finally supervised service provision in the provider trainee’s workplace. Skills were assessed using OSCE during the one-day practicum.

**Results**: Use of the OSCE to assess skills provided valuable information to study team. The performance of provider trainees was high (average 94% correct completion of steps in the OSCE).

**Conclusions**: OSCE was used as a research methodology as part of this pilot study; to date, OSCE has not been integrated into the training approach to be scaled up by FMOH. Uniformly high performance of provider trainees was seen on the OSCE, unsurprising since provider trainees were experienced in providing copper IUD. If and when training is rolled out to providers inexperienced with copper IUD, OSCE may have a more important role to assess skills before service provision. The role of OSCE in design of hybrid digital / in-person training approaches should be further explored in rollout of hormonal IUD and other contraceptive technologies.

## Introduction

Expanding contraceptive method choice, including introducing new contraceptive technologies to health markets, is a critical step in addressing unmet need for family planning. Introduction of new technologies is not without challenge, particularly in low- and middle-income country (LMIC) settings ^1^. The hormonal intrauterine device (IUD), a highly effective long-acting reversible contraceptive method (LARC), has been largely unavailable in sub-Saharan Africa (SSA) as a result of initial high cost of the product, poor understanding of demand for the method, and lack of awareness of the method ^2, 3^. Following years of collaborative effort among a consortium of global stakeholders, the method was added to the procurement catalogs for the U.S. Agency for International Development (USAID) and the United Nations Population Fund (UNFPA) in 2021 ^3^. This, combined with a price reduction of quality-assured hormonal IUD products, led to increased availability of hormonal IUD in LMICs. Several countries in SSA, including Nigeria, are poised to introduce the method on a wider scale ^4^.

Prior to 2019, Nigeria’s efforts to expand access to LARCs were focused on scaling up implants and copper IUD. Since then, Nigeria’s Federal Ministry of Health (FMOH) developed and launched the National Hormonal Intrauterine Device Introduction and Scale-up plan ^5^, a costed implementation plan that outlines policy support and procurement, national training plans with competency assessments, and systems for national scale up. In 2022, implementation of this plan is underway, with quantification of the method for public sector use and training for health care providers. The FMOH and their global partners are exploring efficient and effective approaches to make the strategy a reality and scale up the hormonal IUD. This includes training approaches.

Restrictions during the COVID-19 pandemic disrupted traditional trainings but also opened the door to new approaches, including digital learning. The “new normal” for training approaches, following the COVID-19 pandemic, increasingly includes use of remote technologies ^6^. As digital technologies are introduced into clinical training, corresponding means of assessing clinical competencies must be developed. This study measured competence using an Objective Structured Clinical Examination (OSCE).

The OSCE approach to assessing skills was introduced by Harden in 1975 ^7, 8^, and has been used widely in health sciences and medical education for certification, licensure and in various assessment settings ^9^. In LMICs, OSCE has been used widely in in-service training, particularly with Helping Babies Breathe ^10^, a training model which aims to improve health care provider skills in providing resuscitation to asphyxiated newborns. In Nigeria, OSCE has been integrated into training assessments for medical students and postgraduate medical doctor trainees in various medical training programs. There is a strong literature base discussing the strengths and drawbacks of applications of OSCE in medical education in Nigeria ^11– 16^. While multiple studies exist which look at the use of remote applications of OSCE ^17– 19^, little has been written on the use of OSCE integration into digital training courses, either for hormonal IUD or more broadly.

A study of a hybrid digital and in-person training for hormonal IUD services for health care providers experienced in copper IUD provision was conducted in Nigeria from April to December 2021 (publication under review). The study evaluated feasibility, acceptability, competency, and knowledge gains of health care providers taking the training package. The current article describes the experience of using OSCE as part of the training model. This paper will be useful to policy-makers and program implementers in Nigeria and similar settings who are either planning training programs for hormonal IUD, or considering competency assessment approaches within design of digital training or hybrid digital / in-person training. 

## Methods

### Study Setting

Supported by the Nigeria FMOH, this study was conducted with private and public sector health care providers in Enugu, Kano, and Oyo states in Nigeria. States were chosen in consultation with the state and federal MOH, based on state leadership interest in scaling up hormonal IUD and the presence of Society for Family Health (SFH) franchise facilities and public sector health facilities able to participate in training. Study leadership was provided by FMOH, Population Services International (PSI), and FHI 360. The implementing partner in Nigeria was SFH, a Nigerian non-governmental organization (NGO) working in partnership with communities, government, donors and the private sector for universal health coverage and social justice of all Nigerians. SFH runs a franchise of health facilities (Healthy Family Network) through which it provides family planning and reproductive health services and other health interventions across the country.

### Training Content

The training covered a refresher of other FP methods as well as information on counseling, insertion, and removal of the hormonal IUD. Divided into 13 modules and hosted on a web-based platform called Kaya, the training comprised roughly six hours of content presentation in the form of text, videos, and audio accompaniment, with quizzes interspersed. During the period in which trainees were taking the digital training, WhatsApp support groups for each state were formed and two live sessions were held per state to help with any technological or clinical questions which arose and to offer general support to trainees.

### Training model

The trainees, all health care providers experienced with provision of copper IUD working in health facilities in Enugu, Kano and Oyo states, took the digital training from September–October 2021. Participants had a three-week period to take the digital course which included training slides, videos as well as knowledge assessments. A WhatsApp support group was run concurrently with the digital training component. Following completion of the digital didactic training, trainees came to a one-day, in-person practicum using mannequins. At the close of the in-person practicum, trainees participated in an OSCE assessment. In October 2021, two OSCE assessments with roughly ten provider trainees were held per state (n= 6 OSCE events), to accommodate COVID-19 related restrictions which limited the size of in-person gatherings. In the six weeks after returning to their health facility of employment, the trainees provided hormonal IUDs to at least three clients under supervision of a mentor. The hormonal IUD service provision was evaluated using the same checklist as the OSCE (the FMOH national assessment for provision of hormonal IUD). A passing grade for all three clients (not reported here) was required to receive certification to provide hormonal IUD services. 

### Participants

The trainees were health care providers working in private / SFH and public sector health facilities in participating states. Providers were purposively selected for the training from SFH and public sector facilities based on being a LARC-trained provider and being willing to undertake the training. The selection of LARC-trained providers was inherent in the training design model. IUD service provision in Nigeria is limited to doctors, nurses, midwives, and Senior Community Health Extension Workers (SCHEWs). Trainees who had completed the online didactic training were invited to the one-day practicum using models and OSCE assessments. Trainees had to consent to be part of the OSCE.

### OSCE assessment

Following the digital didactic training, each state convened two clinical practicum events which included OSCE assessment. OSCE assessments were completed in-person, with 10 trainees, one OSCE assessor and one SFH staff attending to assist with logistics and ensure that the OSCE was conducted according to study protocol. Consent was obtained before the trainee commenced the OSCE. 

**Figure 1.**
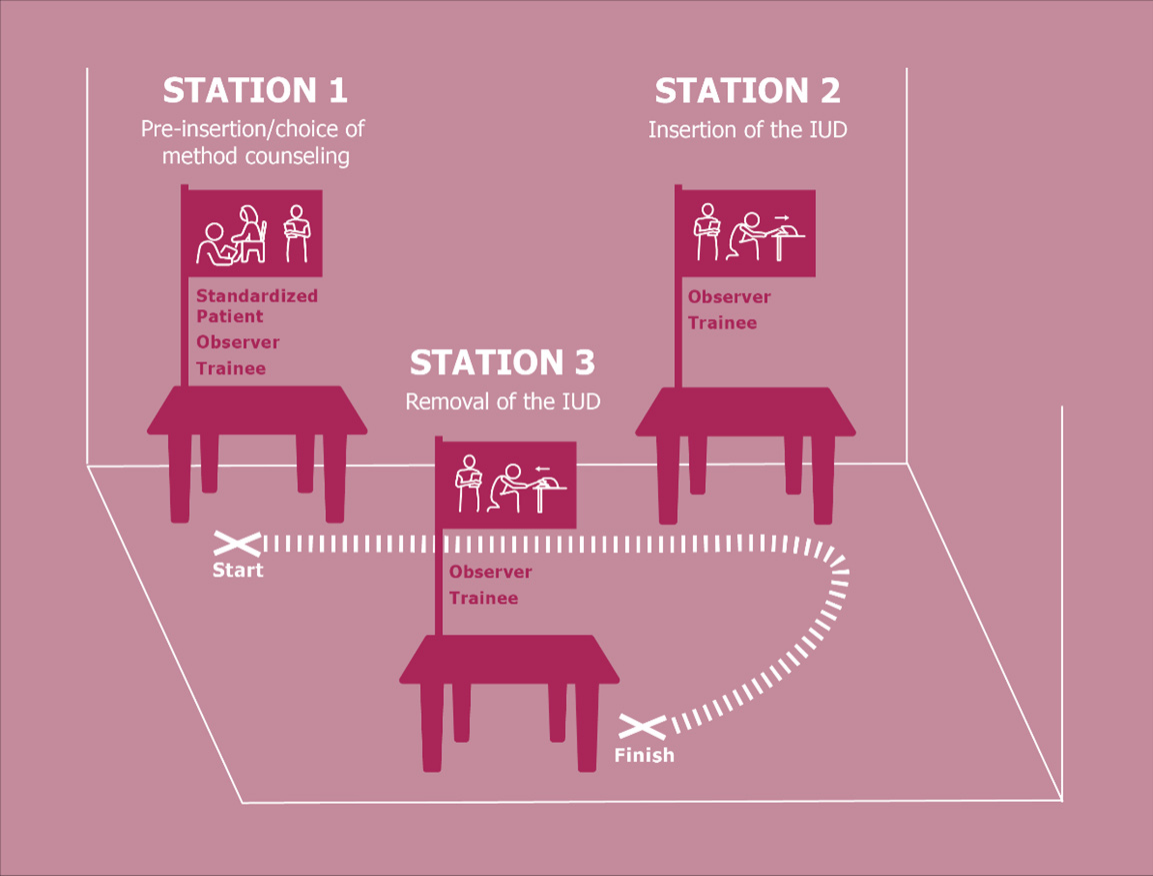
Map of OSCE stations.

**Figure 2.**
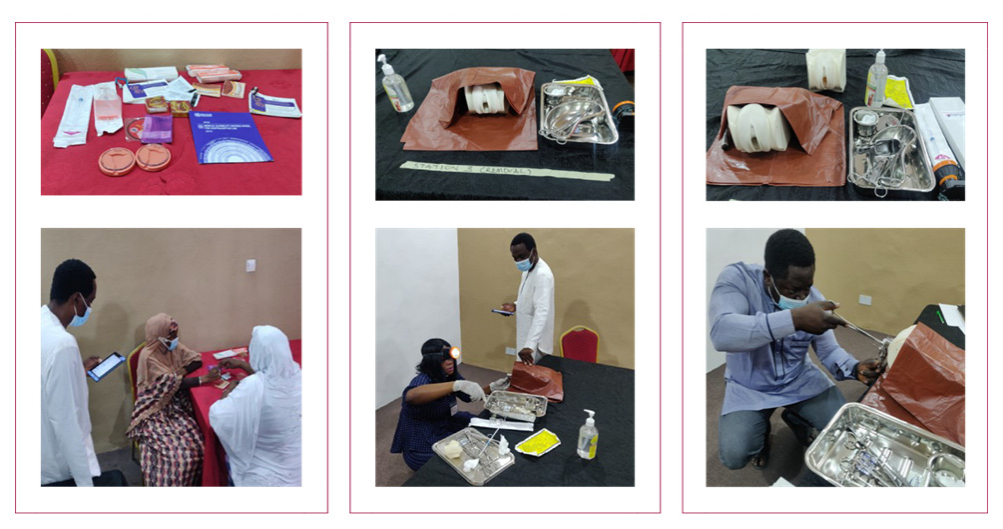
The three OSCE stations with trainees, Kano, Nigeria. Photo credit - Ezechukwu Nwokoma

The OSCE assessment was divided into three stations ( Figure 1, Figure 2) which covered pre-insertion/choice of method counseling (Station 1), insertion of the IUD (Station 2), and removal of the IUD (Station 3). A standardized patient (a person who had been familiarized with a standard scenario and acted as a client) was used for the counseling station.

Master trainers who served as OSCE assessors participated in a two-day training in June 2021 to standardize their scoring on the OSCE. These master trainers came from public tertiary level hospitals and were selected by the FMOH and SFH. Inter-rater reliability between assessor master trainers was conducted by comparing assessment scores of the same event and discussing any discrepant scores until there was complete agreement between assessors. Rounds of inter-rater reliability assessments showed increasing level of agreement between OSCE assessors until complete agreement was achieved. The OSCE assessment tool (see supplemental materials) used the FMOH-approved competency assessment checklist, with slight modifications to reflect the simulation setting. Data were collected by OSCE assessors using tablets and were uploaded from the tablets onto a secure server.

### Scoring and Analysis of OSCE

The FMOH approved the OSCE assessment tool, which was drawn from the Training Resource Package co-developed by a consortium led by USAID, the World Health Organization (WHO), and UNFPA ^20^, the FMOH’s national competency assessment for hormonal IUD (under development), and from PSI’s clinical supervision checklists. The checklist contains critical and non-critical steps. OSCE scores were based on a checklist of 62 items, calculated as a percent of the total points possible. A single point was assigned for each step correctly performed. A minimum score of 80% for non-critical steps and 100% for critical steps was needed to pass. Thus, if a trainee incorrectly completed a critical step, the trainee failed the station. In case a trainee failed, remediation was offered and the trainee was allowed to attempt the station again.

We calculated the mean, median, and range of the scores for each OSCE station and overall, the proportion of providers achieving a passing score of 80% with all critical completed correctly.

Descriptive analyses (means, medians, ranges, and standard deviations) and 95% confidence intervals (CI) were calculated. 

## Ethical oversight

This study was reviewed by FHI 360’s Office of International Research and Ethics (OIRE) and determined exempt (1735182-1). The study was also reviewed by the National Health Research Committee of Nigeria (NHREC) and was approved (NHREC/01/01/2007-03/06/2021). Written informed consent was obtained from all study participants before enrollment in the study, for use of OSCE scores.

## Results

A total of 62 health care providers from Enugu, Kano and Oyo States completed the digital didactic training and took part in the OSCEs. Out of 62 participating trainees, all 62 consented to having their OSCE scores used for research purposes and 60 consented to having demographic information used. Of the 60 trainees whose OSCE scores are presented, 50% were nurses; 33% were midwives; 12% were community health workers and 5% were doctors ( Table 1). The trainees also represented both private and public sectors: 60% of the health care providers were employed in the public sector, 25% in the private sector, and 15% in both public and private sector facilities.

**Table 1.** Demographic characteristics of trainees.

Demographic characteristic	n (%)
	n=60 *
Cadre **	
Nurse	30 (50)
Midwife	20 (33)
Community Health Officer (CHO)	5 (8)
Community Health Extension Worker (CHEW)	2 (3)
Doctor	3 (5)
Sector of Employment	
Public	36 (60)
Private	15 (25)
Both public and private	9 (15)
Average age in years (range)	48 (21, 65)
Gender	
Male	4 (7)
Female	56 (93)
Years of experience in current role	
Under five years	4 (7)
Over five years	56 (93)
Currently providing IUD services (either insertions or removals)	55 (92)
Had previous digital training experience	15 (25)

*While 62 trainees participated in the OSCE, only 60 consented to having their demographic information shared **Contains missing values

### OSCE scores

**Figure 3.**
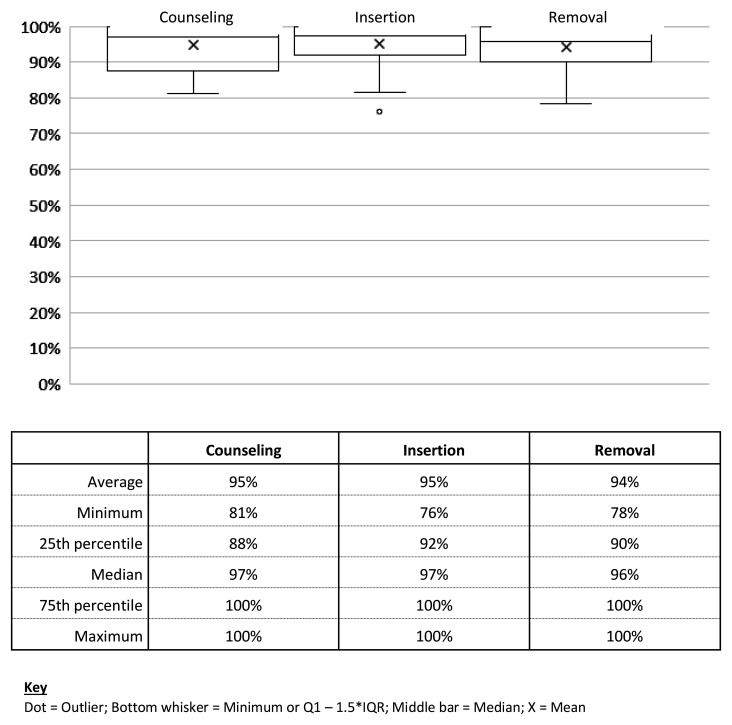
Averages, medians and percentile scores on OSCE.

Of the 62 trainees who took the OSCE, the mean score (combining all three stations) was 94% (95% on the counseling Station, 95% on the insertion Station and 94% on the removal Station) ( Figure 3). Two people “failed” the OSCE assessment, missing critical steps in the insertion station. These initial failures were not due to low scores, rather, in all three cases, the trainee missed at least one critical step ( Table 4).

In the counseling station, most steps were completed correctly by all participants ( Table 2). The lowest scoring steps were: “Describes the medical assessment required before IUD insertion, as well as the procedures for IUD insertion and removal,” correctly performed by 75% of trainees; “Assessed the woman’s knowledge of IUD,” correctly performed by 80% of trainees; and “Helps her to make a plan to manage potential changes to her bleeding,” correctly performed by 82% of trainees.

**Table 2.** OSCE counseling station scores (n=62).

Step Description	Correctly performed the step	
	N	%
Is respectful, sympathetic, and maintains client’s privacy and confidentiality throughout interaction	62	100%
Applies no unnecessary medical or administrative restrictions that exclude youth or other clients from accessing any method	62	100%
Encourages her to ask questions. Provides additional information and reassurance, as needed	56	90%
**Respects client’s choice of family planning method and does not try to coerce/** **pressure her to use one method over another**	**62**	**100%**
Assesses her knowledge of the IUD	49	79%
**Explains how the IUD works**	**62**	**100%**
**Explains the benefits and limitations of the IUD**	**62**	**100%**
**Comprehensively explains possible side effects**	**62**	**100%**
**Comprehensively explains possible changes in bleeding, including amenorrhea**	**62**	**100%**
Helps her to make a plan to manage potential changes to her bleeding (e.g., need for menstrual hygiene products)	50	81%
Explains that the IUD does not protect against sexually transmitted infections (STIs), including HIV.	58	94%
**Explains condom use for dual protection to prevent STIs/HIV**	**62**	**100%**
Describes the medical assessment required before IUD insertion, as well as the procedures for IUD insertion and removal	47	76%
**Ensures client safeguarding throughout the entire experience of care**	**62**	**100%**
**Once the woman has chosen to use the IUD, determines the client is not ** **pregnant (acts accordingly if she is)**	61	98%
**Ensures that equipment and supplies are available and ready to use**	**62**	**100%**

*Bold indicates essential steps*

In the insertion station, most steps were completed correctly by all participants ( Table 3). The lowest scoring steps were: “Ensures that equipment and supplies are available and ready to use,” correctly performed by 80% of trainees; “Reviews the insertion procedure again and keeps the client informed of what is happening throughout the procedure,” correctly performed by 80% of trainees and “Tells the client what examinations are being performed and their purpose, asks her if she has any questions,” correctly performed by 83% of trainees. One participant failed the essential steps “Makes appropriate decision on proceeding with insertion and communicates with client” and “Applies gentle traction on the tenaculum before advancing the IUD up into the uterine cavity”, while another participant failed “Uses HLD (or sterile) sharp Mayo scissors to cut the IUD strings to a 3 cm to 4 cm length, while the ends of the strings are still in the inserter tube.” Both participants repeated the station and passed on the second attempt.

**Table 3.** OSCE insertion station scores (n=62).

Step Description	Correctly performed the step	
	N	%
**Determines the client is not pregnant**	**62**	**100%**
Ensures that equipment and supplies are available and ready to use	49	79%
Tells the client what examinations are being performed and their purpose, asks her if she has any questions	51	82%
Has the client empty her bladder and wash her perineal area	58	94%
Helps the client onto the examination table	53	85%
**Washes hands**	**62**	**100%**
**Puts clean examination gloves on both hands**	**62**	**100%**
**Performs abdominal exam**	**62**	**100%**
**Performs vaginal exam**	**62**	**100%**
**Performs a bimanual pelvic exam**	**62**	**100%**
Removes and disposes of gloves correctly, then puts new clean examination gloves on both hands.	54	87%
**Performs a speculum exam**	**62**	**100%**
**Makes appropriate decision on proceeding with insertion and communicates with client**	**61**	**98%**
Reviews the insertion procedure again and keeps the client informed of what is happening throughout the procedure	50	81%
Cleanses the cervical os and vaginal wall with antiseptic	60	97%
**Gently grasps the cervix with an HLD (or sterile) tenaculum and applies gentle traction**	**62**	**100%**
Inserts the HLD (or sterile) sound using the no-touch technique, and measures the uterine depth	60	97%
Loads the IUD in its sterile package, using the no-touch technique	61	98%
Sets the upper edge of the depth gauge (flange) on the loaded IUD inserter to correspond to the uterine depth as measured by the sound	59	95%
Gently applies traction on the tenaculum to straighten the alignment of the cervical canal and the uterine cavity	60	97%
**Slides the loaded IUD insertion tube through the cervical canal until the upper edge of ** **the flange is 1.5 cm to 2.0 cm from the cervical os**	**62**	**100%**
Releases the hold on the tenaculum	58	94%
Holds the inserter tube with the dominant hand and the rod with the non-dominant hand	57	92%
Holds the rod still and pull the inserter tube back until it reaches the edge of the second (bottom) indent on the rod	59	95%
**Waits for 10–15 seconds for the arms of the IUD to open fully while holding the insertion ** **tube and the rod steady**	**62**	**100%**
**Applies gentle traction on the tenaculum before advancing the IUD up into the uterine ** **cavity**	**61**	**98%**
**Advances the inserter tube (with the rod inside) up into uterine cavity to the fundus, ** **until a slight resistance is felt (the flange is at the cervical opening)**	**62**	**100%**
Holds the rod stable with one hand and pull the inserter tube back to the ring of the rod with the other hand	60	97%
While holding the inserter tube, first withdraws the rod from the inserter tube and then withdraws the inserter tube until the tube is 3-4 cm away from the cervical os.	62	100%
**Uses HLD (or sterile) sharp Mayo scissors to cut the IUD strings to a 3 cm to 4 cm length, ** **while the ends of the strings are still in the inserter tube**	**61**	**98%**
Gently removes the tenaculum and process according to infection prevention protocols	62	100%
Examines the cervix for bleeding; if no bleeding, gently removes the speculum and process according to infection prevention protocols	51	82%
Asks the client how she is feeling, tells her that the procedure is complete and observes her for at least 15 mins	55	89%
Provides post-insertion instructions	55	89%
**Before removing the gloves, processes instruments according to infection prevention ** **protocol**	**62**	**100%**
Properly disposes of waste materials	59	95%
Processes gloves according to recommended infection prevention practices	59	95%
Washes hands thoroughly with soap and water and dries them	61	98%

*Bold indicates essential steps*

In the removal station, most steps were completed correctly by all participants ( Table 4). The lowest scoring steps were: “Reviews the client’s reproductive goals and the need for STI protection, and counsels her,” correctly performed by 80% of trainees; and “Ensures that equipment and supplies are available and ready to use,” correctly performed by 83% of trainees.

**Table 4.** OSCE removal station scores (n=62).

Step Description	Correctly performed the step	
	N	%
Asks the woman her reason for having the IUD removed, mentioning that if side effects are an issue the provider could help manage those	58	94%
**Does not pressure the client to continue using the IUD, respects her right to use or not ** **use contraception and does not refuse or delay her request**	**62**	**100%**
Reviews the client’s reproductive goals and the need for STI protection, and counsels her	50	81%
Determines whether she will have another IUD inserted immediately, start a different method, or neither	52	84%
Explains what will happen during the removal procedure	54	87%
Ensures that equipment and supplies are available and ready to use	52	84%
Asks client to empty her bladder and wash her perineal area	57	92%
Helps the client onto the examination table	56	90%
**Washes hands thoroughly with soap and water and dries them**	**62**	**100%**
**Put clean examination gloves on both hands**	**62**	**100%**
Reminds client of the removal procedure and explains what is happening throughout the removal procedure. Asks client to tell if she feels any pain during the procedure. Reassures that some discomfort is to be expected.	56	90%
Gently inserts the HLD (or sterile) speculum to visualize the strings, and cleanses the cervical os and vaginal wall with antiseptic	62	100%
Alerts the client prior to removing the IUD	55	89%
Grasp the IUD strings close to the cervix with an HLD (or sterile) long artery or Kelly forceps	62	100%
**Applies steady but gentle traction, pulling the strings gently but firmly, to remove the ** **IUD. Does not use excessive force**	**62**	**100%**
Shows the IUD to the client	57	92%
Before removing gloves, disposes of the IUD following safe infection prevention practices	60	97%
Gently removes the speculum, and processes instruments according to infection prevention protocols.	62	100%
Asks the client how she is feeling.	58	94%
If the woman is starting a different method, provides the information she needs to use it safely and effectively [and a back-up method, if needed]	59	95%
**Before removing the gloves, processes instruments according to infection prevention ** **protocol**	**62**	**100%**
Properly disposes of waste materials	60	97%
Washes hands thoroughly and dries them	61	98%

*Bold indicates essential steps*

## Discussion

While digital trainings may be a cost-effective option for health care providers in LMICs, there is limited evidence about the effectiveness, feasibility, and impact of the training on both health systems and at the individual level ^21^. This study assessed competency scores following a hybrid digital / in-person training that aimed to expand high-quality hormonal IUD services in Nigeria. In the process of looking at competency scores and processes associated with using OSCE as a research assessment methodology, we gained useful insight into the overall training model and process, some of which is unique to hormonal IUD. 

For purposes of the pilot study described here, OSCE was included. However, the training model evaluated, which has been endorsed by the FMOH to be included in national scale up of hormonal IUD in Nigeria ^5^, uses supervised provision of service rather than OSCE to establish competency, for both cost savings and training efficiency reasons. With the group enrolled in the study (health care providers who had previous experience in providing copper IUDs group), it would have been surprising to see low level of competency in provision of hormonal IUD services, particularly insertions and removals, since those skills do not vary between the hormonal and copper IUD. Given extremely high competency seen among these experienced providers, OSCE as a competency assessment approach may not be necessary in the non-research (i.e. training rollout) context. The experienced background of the trainees in IUD service provision may explain differences between the findings in this study and one which assessed provider skills in nine countries in sub Saharan Africa and Asia following training on obstetric skills, which documented higher skills gains using the OSCE assessment (23 – 35%) ^22^. An outstanding question is whether the OSCE would serve as an important training component by identifying providers not yet sufficiently skilled in hormonal IUD services among inexperienced providers. If hormonal IUD services are to be scaled up to include providers who are not already trained and experienced in providing LARCs, there is a strong argument for vetting skills using OSCE before trainees proceed to supervised service provision.

While digital trainings may save time and resources compared to didactic classroom-based elements, clinical trainings, including hormonal IUD training, will generally benefit from some in-person components. There is precedent for including OSCE as a training approach to improve the hormonal IUD training model: OSCE is a proven approach to both teaching and evaluation of competency ^8, 9^. In Nigeria, OSCE has shown to compare favorably with other assessment methodologies for medical students ^12, 13, 15, 16^. In the United States, standardized patients at OSCE stations have been used to assess resident doctors’ ability to counsel vaccine-hesitant patients ^23^, and medical students’ ability to counsel patients with obesity ^24^. OSCE has been used to improve communication, interpersonal and counseling skills ^25^. Future training models for hormonal IUD scale up may want to explore use of OSCE as a training tool rather than (or in addition to) as an assessment tool. Building competency may be even more important in the context of digital training where trainers lack the ability to interact with trainees face-to-face to get a sense of their communication skills and fluency with the content. Additionally, there may be applications for assessing retention of health care providers’ skills and knowledge over time, and the acquisition of skills and knowledge among a larger pool of providers as hormonal IUD is scaled up in Nigeria.

As training for hormonal IUD expands in Nigeria and globally, future studies may want to measure outcomes of training, rather than the focus on model which guided the current study. In Ghana, Kenya, and India, for example, perinatal mortality was tracked following Helping Babies Breathe training ^26, 27^. An evaluation of the hybrid digital / in-person training model should ideally include outcomes, such as post-training behavior of health care providers, or provision of hormonal IUD services where health care providers were trained ^28^. A focus on outcomes moves the training further along the levels described by Kirkpatrick’s model for evaluation of training programs, from Reaction and Learning to Behavior and Results ^29^.

This study had some limitations. A specific limitation of the OSCE approach was that the stations were not timed, which may have resulted in higher scores than would have otherwise been seen. Further, no baseline OSCE was performed to assess changes in clinical performance as a result of the training. Additionally, generalizability is limited due to the small number of health care providers trained and the purposive selection of the three states.

## Conclusion

This study presents findings from OSCE assessment in a study of a digital / in-person training model for hormonal IUD in Nigeria. The uniformly high scores of these experienced LARC providers in the study makes it questionable whether OSCE should be incorporated into a scaled-up training model. However, if the training is extended to include “LARC-inexperienced” health care providers, the OSCE assessment may become important as a means to assess competency before trainees provide supervised services to clients. Future evaluations would be helpful in investigating the role of OSCE in training for health care providers who are inexperienced at providing LARCs. Finally, future studies on digital or hybrid digital / in-person training should look to incorporate outcomes of training into design, to further the agenda of transforming training into outcomes and impacts for those we are trying to reach with contraceptive technologies.

## Data Availability

Harvard Dataverse. “Hybrid digital hybrid training approach for hormonal IUD in Nigeria (R4S study 2.8)", DOI: https://doi.org/10.7910/DVN/4PHEUT This project contains the following underlying data: OSCE Checklist.pdf. (Data collection instrument) OSCE.tab. (OSCE data) Data are available under the terms of the Creative Commons Zero "No rights reserved" data waiver (CC0 1.0 Public domain dedication).
