# Supplementary material for: Use of Objective Structured Clinical Examination (OSCE) in a hybrid digital / in-person training for hormonal IUD in Nigeria: findings and applications of the approach
Source: Gates Open Res. 2023 Sep 12;7:120. [Version 1] doi: 10.12688/gatesopenres.14695.1 (PMC10673859; doi:10.12688/gatesopenres.14695.1)
Supplement: Peer review history

# Reviewer report: Reviewer response for version 1

**DOI:** 10.21956/gatesopenres.16020.r35126

**Published:** 2023-11-24

**Authors:** Akanni Akinyemi

**Affiliations:** 1 Department of Demography and Social Statistics, Obafemi Awolowo University, Ife, Osun, Nigeria

I found the manuscript very interesting and of value addition in addressing issues around the supply side of IUD in Nigeria.

 There are few comments that may be useful:

1. There is no information on the prior knowledge level of the participants. Are they exposed to something completely new to them?
2. Because of the small sample size, it is important to provide more detailed information about the average number of clients they provide service to prior and after the training.

Is the work clearly and accurately presented and does it cite the current literature?

Yes

If applicable, is the statistical analysis and its interpretation appropriate?

Yes

Are all the source data underlying the results available to ensure full reproducibility?

Yes

Is the study design appropriate and is the work technically sound?

Yes

Are the conclusions drawn adequately supported by the results?

Yes

Are sufficient details of methods and analysis provided to allow replication by others?

Yes

Reviewer Expertise:

Global health, Demography

I confirm that I have read this submission and believe that I have an appropriate level of expertise to confirm that it is of an acceptable scientific standard.

# Reviewer report: Reviewer response for version 1

**DOI:** 10.21956/gatesopenres.16020.r35128

**Published:** 2023-10-11

**Authors:** Julia Bluestone

**Affiliations:** 1 Jhpiego Corporation, Baltimore, Maryland, USA

Thanks for the chance to review this well design study and well-written article. It is clearly written, with strong and objective validation via supporting resources. See a few comments: 

**Under the Introduction section:**

 “As digital technologies are introduced into clinical training, corresponding means of assessing clinical competencies must be developed. This study measured competence using an Objective Structured Clinical Examination (OSCE).”— *I suggest strengthening this statement to reinforce the need to demonstrate that blended approaches can be just as effective to improve skill competency, at potentially lower costs, thus use of OSCE is critical to demonstrate similar learning outcomes can be achieved. OSCE doesn’t really correspond to digital tech, rather it is a robust measure of skill competency that is well accepted in the education field. (as you nicely reinforce in the para that follows)*

**Under ‘methods’**

 Training model: *please specify-was there a schedule or structure for the asynchronous content (roughly six hours) over the three week period? Or was it completely up to the learner to complete before the in-person, synchronous event? Please provide a bit more detail on the structure for the independent, asynchronous learning.*

 OSCE assessment- *nice job speaking to how you ensured interrater reliability.*

**Discussion:**

 “While digital trainings may save time and resources compared to didactic classroom-based elements, clinical trainings, including hormonal IUD training, will generally benefit from some in-person components.” *- I would explain why-and hone it a little bit-you could say something to the effect of ‘for clinical trainings that require psychomotor skills that convey risk of harm to clients, there is benefit from skills practice and supervision in a live, simulated session to ensure skill competency and reduce risk of harm to clients.' do as you wish, but I think this point should be made*

**Conclusion:**

 “However, if the training is extended to include “LARC-inexperienced” health care providers, the OSCE assessment may become important as a means to assess competency before trainees provide supervised services to clients. Future evaluations would be helpful in investigating the role of OSCE in training for health care providers who are inexperienced at providing LARCs.” *– I agree 100% here, and would suggest that you strengthen this language a bit-any time we are building psychomotor skills that convey potential harm to clients, assessing in simulation using validated measures such as OSCE should be a priority to protect clients. I think ‘may be come important’, in my mind, could be, ‘it is critical’-consider strengthening this conclusion*

Is the work clearly and accurately presented and does it cite the current literature?

Yes

If applicable, is the statistical analysis and its interpretation appropriate?

I cannot comment. A qualified statistician is required.

Are all the source data underlying the results available to ensure full reproducibility?

Yes

Is the study design appropriate and is the work technically sound?

Yes

Are the conclusions drawn adequately supported by the results?

Yes

Are sufficient details of methods and analysis provided to allow replication by others?

Yes

Reviewer Expertise:

Issues regarding improving health worker learning and performance, pre-service education.

I confirm that I have read this submission and believe that I have an appropriate level of expertise to confirm that it is of an acceptable scientific standard.
